# Managing Hypercapnia in Patients with Severe ARDS and Low Respiratory System Compliance: The Role of Esophageal Pressure Monitoring—A Case Cohort Study

**DOI:** 10.1155/2015/385042

**Published:** 2015-01-28

**Authors:** Arie Soroksky, Julia Kheifets, Zehava Girsh Solomonovich, Emad Tayem, Balmor Gingy Ronen, Boris Rozhavsky

**Affiliations:** ^1^Intensive Care Unit, E. Wolfson Medical Center, 62 HaLohamim Street, P.O. Box 5, 58100 Holon, Israel; ^2^Sackler Faculty of Medicine, Tel Aviv University, Tel Aviv, Israel

## Abstract

*Purpose*. Patients with severe acute respiratory distress syndrome (ARDS) and hypercapnia present a formidable treatment challenge. We examined the use of esophageal balloon for assessment of transpulmonary pressures to guide mechanical ventilation for successful management of severe hypercapnia. *Materials and Methods*. Patients with severe ARDS and hypercapnia were studied. Esophageal balloon was inserted and mechanical ventilation was guided by assessment of transpulmonary pressures. Positive end expiratory pressure (PEEP) and inspiratory driving pressures were adjusted with the aim of achieving tidal volume of 6 to 8 mL/kg based on ideal body weight (IBW), while not exceeding end inspiratory transpulmonary (EITP) pressure of 25 cm H_2_O. *Results*. Six patients with severe ARDS and hypercapnia were studied. Mean PaCO_2_ on enrollment was 108.33 ± 25.65 mmHg. One hour after adjustment of PEEP and inspiratory driving pressure guided by transpulmonary pressure, PaCO_2_ decreased to 64.5 ± 16.89 mmHg (*P* < 0.01). Tidal volume was 3.96 ± 0.92 mL/kg IBW before and increased to 7.07 ± 1.21 mL/kg IBW after intervention (*P* < 0.01). EITP pressure before intervention was low with a mean of 13.68 ± 8.69 cm H_2_O and remained low at 16.76 ± 4.76 cm H_2_O (*P* = 0.18) after intervention. Adjustment of PEEP and inspiratory driving pressures did not worsen oxygenation and did not affect cardiac output significantly. *Conclusion*. The use of esophageal balloon as a guide to mechanical ventilation was able to treat severe hypercapnia in ARDS patients.

## 1. Introduction

Treating acute respiratory distress syndrome (ARDS) patients with lung protective ventilation [1] entails limitations on applied plateau pressure. Patients with excessively low respiratory system compliance may result in markedly low tidal volume and at times even below the recommended 6 to 8 mL/kg of ideal body weight (IBW). This may culminate in hypercapnia and severe respiratory acidosis [2, 3].

Due to the low respiratory system compliance, any attempt to lower PaCO_2_ by increasing alveolar ventilation may require an increase in inspiratory driving pressure, which may expose patients to excessively high plateau pressures.

Thus, in such patients, exercising lung protective ventilation may result in severe hypercapnia and severe respiratory acidosis, consequently leaving us with few treatment options. In such patients the only option to reverse severe respiratory acidosis may require the use of measures that remove CO_2_ extracorporeally [4, 5], while at the same time allowing us to continue and exercise lung protective ventilation.

The use of esophageal balloon with measurement of transpulmonary pressure allows us to partition the respiratory system into its components and thus better direct inspiratory driving pressure and positive end expiratory pressure (PEEP). The aim of this report is to describe six consequent patients with bilateral pneumonia and ARDS, who had excessively low respiratory system compliance and severe hypercapnia with severe respiratory acidosis. The management of these patients was guided by measurement of transpulmonary pressures. Adjustment of inspiratory driving pressure and PEEP based on transpulmonary pressures resulted in a dramatic decrease in PaCO_2_ and thus the avoidance of invasive extracorporeal CO_2_ removal.

## 2. Methods

Patients described in this report were enrolled in a larger ongoing study (ClinicalTrials.gov number NCT01668368), in which esophageal balloon is used to direct adjustments in PEEP and inspiratory driving pressure. This study has been approved by the local ethics committee in accordance with the Declaration of Helsinki, and informed consent was obtained.

For the purpose of this study we have developed inclusion criteria for recruiting patients and for esophageal balloon insertion. The purpose of these inclusion criteria was to select the patients with the most severe respiratory failure with ARDS who would benefit the most from an intervention that is guided by esophageal balloon measurements. ARDS was defined according to the Berlin definition [6].

**Figure 1:**
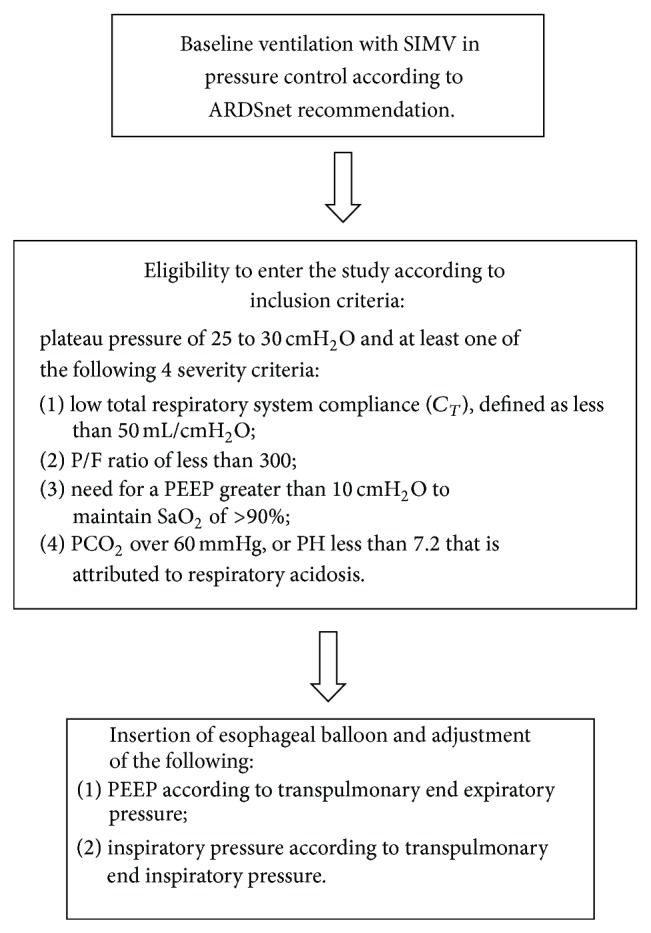
Inclusion criteria for insertion of esophageal balloon and patient recruitment into the study. Once esophageal balloon was inserted, PEEP was adjusted according to end expiratory transpulmonary (EETP) pressure, with the aim of keeping EETP slightly positive. Inspiratory driving pressure was adjusted according to end inspiratory transpulmonary (EITP) pressure, with the aim of achieving tidal volume of 6 to 8 mL/kg IBW, while keeping EITP less than 25 cm H_2_O. PEEP: positive end expiratory pressure. ^*^ARDS was defined according to the Berlin definition [6].

Eligibility criteria (Figure 1) for insertion of esophageal balloon included any patient with acute respiratory failure of any cause who was mechanically ventilated according to the ARDS network guidelines with a prerequisite of high inspiratory driving pressure (plateau pressure of up to 25 to 30 cm H_2_O) and at least one of the following four severity inclusion criteria: (1) low total respiratory system compliance (CT), defined as less than 50 mL/cm H_2_O; (2) P/F ratio of less than 300 mmHg; (3) need for a PEEP greater than 10 cm H_2_O to maintain SaO_2_ of >90%; and (4) PaCO_2_ over 60 mmHg or PH less than 7.2 that is attributed to respiratory acidosis.

For patient enrolment, eligibility criteria had to be met within 24 hours of ICU admission or within 24 hours from commencing mechanical ventilation. Patients with any of the following were excluded from the study: known bronchial asthma or chronic obstructive pulmonary disease (COPD), previous lung or chest wall surgery, previous esophageal surgery, known achalasia or any other esophageal motility or spasm disorder, known or suspected esophageal varices, presence of chest thoracostomy tube that was inserted due to pneumothorax, and any significant chest wall abnormality such as kyphoscoliosis.

## 3. Intervention

Patients were supine and were ventilated by a commercially available ventilator (Avea, CareFusion Inc., CA, USA). The ventilator is supplied with a built-in module allowing the connection of an esophageal balloon catheter for continuous transpulmonary pressure monitoring. Upon fulfillment of inclusion criteria esophageal balloon was inserted. The balloon was first inserted into the stomach to a depth of 60 to 70 cm from the incisors. Thereafter, it was slowly pooled caudally until heart beat could be noticed on the esophageal pressure tracing. For further confirmation of correct esophageal balloon positioning an “occlusion test” was performed. We have modified the original “occlusion test” [7] by inserting a thin pressure recording tracheal catheter capable of measuring pressure in air interface.

**Figure 2:**
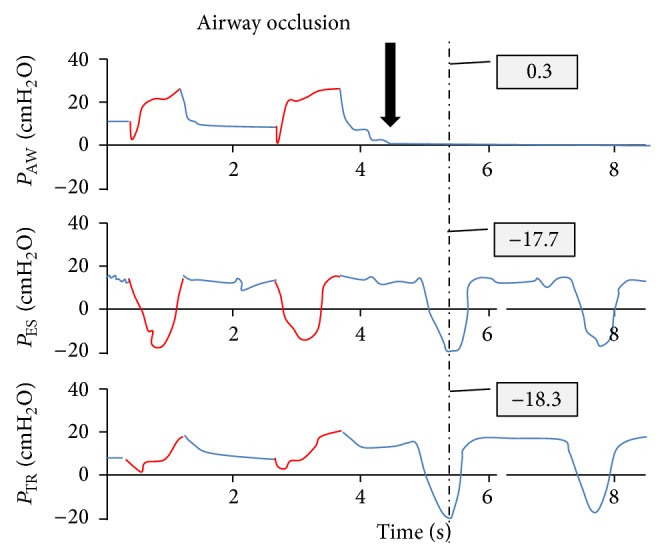
“Occlusion test.” Representative pressure tracing of one of the patients. After the second breath, inspiratory and expiratory ventilator tubing are occluded (bold arrow). The third and fourth inspiratory effort are made against an occluded airway. Airway pressure tracing is occluded and is thus close to zero. However, large negative deflections can be noticed on the esophageal and tracheal pressure tracing, and in this case the values of both are close to unity, thus indicating a proper position of the esophageal balloon. *P*_AW_: airway pressure, *P*_ES_: esophageal pressure, and *P*_TR_: tracheal pressure at the distal end of endotracheal tube and close to the carina.

The tip of the catheter was positioned at the distal end of the endotracheal tube and close to the carina. Thereafter, inspiratory and expiratory tubes of the ventilator were occluded to allow at least two inspiratory efforts to be made against an occluded airway (Figure 2). A correct esophageal balloon position was considered appropriate if the values of esophageal and tracheal pressures during an inspiratory effort against an occluded airway were within 10% of each other.

After verifying an appropriate esophageal balloon placement, plateau pressure was measured by applying an inspiratory hold for 1 to 2 seconds at end inspiration, followed by assessment of transpulmonary end inspiratory and end expiratory pressures. Thereafter, PEEP was adjusted according to end expiratory transpulmonary (EETP) pressure, with the aim of keeping EETP pressure close to zero or slightly positive, while achieving oxygenation target of PaO_2_ of 60 to 90 mmHg, or oxygen saturation of 88 to 95%; inspiratory driving pressure was adjusted according to end inspiratory transpulmonary (EITP) pressure, with the aim of achieving a tidal volume of 6–8 mL/kg (IBW), while at the same time not exceeding EITP pressure of 25 cm H_2_O.

Lung compliance was calculated by dividing tidal volume by end inspiratory transpulmonary pressure, while chest wall compliance was calculated by dividing tidal volume by pleural pressure. All patients were monitored continuously with arterial line, heart rate, blood pressure, oxygen saturation, end tidal CO_2_, and transpulmonary thermodilution technique with continuous cardiac output assessment using arterial pulse contour analysis (PiCCO_2_) (PULSION Medical Systems AG, Munich, Germany).

Statistical analysis was performed using BMDP [8]. We compared all the first values with the second values analysis of variance (ANOVA) with repeated measures.

Due to the small sample size and the relatively large number of comparisons, a *P* value of less than or equal to 0.01 was considered statistically significant.

## 4. Results

Six consecutive patients with severe hypercapnia and a concomitant significant hypoxemia requiring moderate to high PEEP which was set according to the algorithm of ARDSnet guidelines [1] were enrolled. All six patients had bilateral pneumonia with ARDS.

In all patients esophageal balloon insertion was successful and without any complications.

Patient characteristics on recruitment are shown in Table 1. All had high APACHE II scores, with a mean of 31.16 ± 4.75 and a high predicted mortality 69.53 ± 10.98.

All had severe hypoxemia requiring the use of moderate to high PEEP values which was set according to the algorithm of ARDSnet guidelines. Respiratory parameters of individual patients and as a group, before and after intervention, are presented in Tables 2 and 3. The mean P/F ratio on enrollment was 144.25 ± 54.15 mmHg and 158.66 ± 30.11 mmHg (*P* = 0.45) after intervention guided by esophageal balloon measurements. The mean PEEP value on patient enrolment was 13.66 ± 2.16 cm H_2_O and 10.83 ± 5.45 cm H_2_O (*P* = 0.18) after intervention.

The mean PaCO_2_ on patient recruitment was 108.33 ± 25.65 mmHg and decreased to 64.5 ± 16.89 mmHg (*P* = 0.003), one hour after intervention. Mean tidal volume was 3.96 ± 0.92 mL/kg/IBW before and increased to 7.07 ± 1.21 mL/kg/IBW after intervention (*P* < 0.001). After 24 hours, PaCO_2_ blood levels along with all the other respiratory parameters did not change significantly (data not shown).

In five out of the six patients inspiratory driving pressure was increased to 25 cm H_2_O and remained unchanged in one patient.

Assessment of pleural pressure identified very low EITP pressure in all 6 patients. This allowed us to increase inspiratory driving pressure in 4 out of 6 patients. In the remaining two patients, an unexpected positive EETP was found. Consequently, lowering PEEP in these two patients resulted in a significant improvement in alveolar ventilation and a decrease in PaCO_2_ from 140 to 96 mmHg and from 81 to 50 mmHg, respectively (patients 5 and 6 in Table 2).

Concomitantly, lowering PEEP in these two patients resulted also in an increase in cardiac index from 1.8 to 2.6 L/min/m^2^ and from 3.2 to 3.95 L/min/m^2^, respectively (patients 5 and 6 in Table 2).

In all patients, intervention guided by esophageal balloon measurements which included raising inspiratory driving pressure in five patients and lowering PEEP in two patients did not affect oxygenation significantly; mean P/F ratio was 144.25 ± 54.15 mmHg before and 158.66 ± 30.11 mmHg after intervention (*P* = 0.45). However, lowering PEEP in patients 5 and 6 has slightly improved P/F ratio from 130 to 135 mmHg and from 151 to 166 mmHg, respectively.

As expected from the severity and from the predicted mortality, only 2 out of 6 patients were alive at 28 days. The direct cause of death in all four patients was sepsis with multiorgan failure.

## 5. Discussion

The use of esophageal balloon for assessment of pleural pressure has largely been an investigational tool [9–11]. However, in recent years, esophageal balloon, although not yet widely available and accepted, has become commercially available. Studies published in recent years [12–14] reported on the successful use of esophageal balloon and its feasibility. The reports of Talmor et al. [12, 13] demonstrated how ventilation guided by esophageal balloon improved oxygenation. One report even showed that ventilation guided by esophageal balloon may avert the need for extracorporeal membrane oxygenation (ECMO) in some patients with severe ARDS [14].

The interpretation of esophageal balloon measurements may be compounded by factors such as inappropriate position of the balloon in a way that will cause false readings. However, in our study proper esophageal balloon placement was verified in all patients by the occlusion test. Furthermore, during assessment of pleural pressure the weight of mediastinal structures such as the heart has to be accounted for. Washko et al. [15] studied 10 healthy subjects and showed that mediastinal structures added 3 ± 2 cm H_2_O to the measured esophageal pressure. However, it should be noted that with increasing airway pressure there is a possibility for a concomitant decrease of superimposed pressure [16]. This could partly be explained by a possible shift of blood out of the thorax with increasing airway and pleural pressure.

Talmor and his group used a similar correction in two recent reports [12, 13]. They subtracted 3 cm H_2_O for the possible weight of the heart and another 2 cm H_2_O to correct for the effects of air volume within the esophageal balloon catheter.

Another recent report compared two methods of correction of measured esophageal pressure and found that correcting esophageal pressure measurements obtained at relaxation volume of the respiratory system is more accurate than using the 5 cm H_2_O offset to account for the weight of mediastinal structures [17].

Thus, the appropriate correction factor that should be applied when we interpret esophageal pressure measurements is still controversial. Furthermore, the main goal of setting up appropriate PEEP is to minimize cyclic recruitment and derecruitment.

In line with this theory, preventing cyclic recruitment and derecruitment is probably best achieved when PEEP is set to attain a slightly positive EETP pressure. For these reasons and for the sake of simplicity we chose not to subtract from the measured esophageal value.

In this report we describe six patients with acute respiratory failure (Table 1) who also had low respiratory system compliance and at the same time severe hypercapnia with severe respiratory acidosis. All six patients had bilateral pneumonia with ARDS. In 3 out of the 6 hypoxemic patients, PEEP was set to 15 cm H_2_O (guided by the ARDSnet guidelines); thus in order not to exceed a plateau pressure of 30 cm H_2_O, inspiratory driving pressure in these 3 patients could not be more than 15 cm H_2_O. This resulted in very low tidal volume and consequently in severe hypercapnia and respiratory acidosis. A fourth patient had a starting PEEP of 12 cm H_2_O, and as in the previous 3 patients a similar inspiratory driving pressure was still inadequate in terms of alveolar ventilation and resulted in hypercapnia as well.

**Figure 3:**
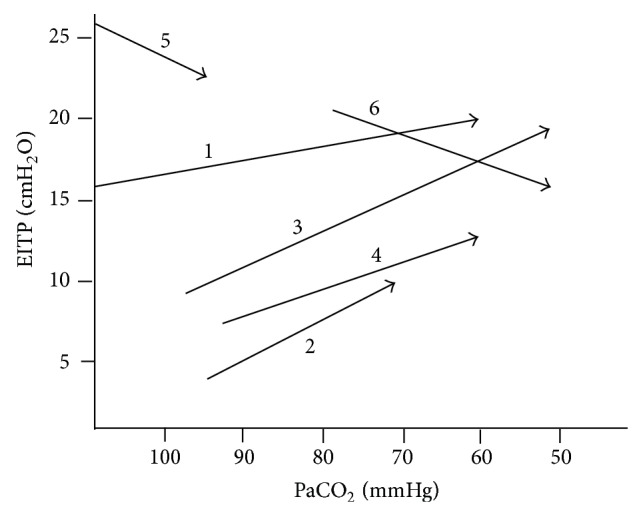
Relationship between EITP and PaCO_2_. Patients 1 to 4 had a low EITP which resulted in extremely low tidal volumes. The increase in inspiratory driving pressure increased EITP with resulting increase in tidal volumes and eventual decrease in PaCO_2_. However, patients 5 and 6 had a high EITP due to inappropriately high PEEP pressure reflected by the high EETP. Consequently, PEEP was lowered to obtain a close to zero EETP. The resulting decrease in EETP resulted also in a decrease in EITP, both of which resulted in a significant decrease in PaCO_2_ from 140 to 96 and from 81 to 50 mmHg in patients 5 and 6, respectively. EITP: end inspiratory transpulmonary pressure, EETP: end expiratory transpulmonary pressure, and PEEP: positive end expiratory pressure.

**Figure 4:**
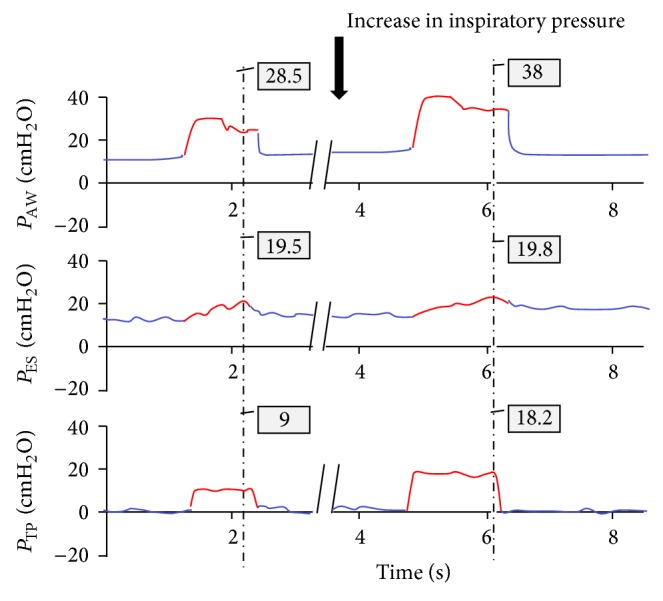
Representative pressure tracing of patient number 3. Inspiratory driving pressure was increased from 15 to 25 cm H_2_O. Although plateau pressure increased from 28.5 to 38 cm H_2_O, end inspiratory transpulmonary (EITP) pressure did not exceed 18.2 cm H_2_O (lower pressure tracing). The increased EITP pressure resulted in a marked improvement in alveolar ventilation and consequent reduction in PaCO_2_ from 99 to 53 mmHg, while at the same time keeping EITP pressure well within acceptable limits. *P*_AW_: airway pressure, *P*_ES_: esophageal pressure, and *P*_TP_: transpulmonary pressure.

Four patients were found to have EETP pressure close to zero, and therefore raising PEEP further was not necessary. However, EITP pressure was low (at 16.6, 2.7, 9, and 7.6 cm H_2_O) (Figure 3). Thus, in spite of plateau pressure of close to 30 cm H_2_O, these low values of EITP allowed us to increase inspiratory driving pressure from 15 to 25 cm H_2_O (on top of 15 cm H_2_O of PEEP) (Figure 4). By doing so, plateau pressure exceeded 30 in all 4 patients. However, EITP pressure remained acceptable and well below the upper safety limit of 25 cm H_2_O.

Not surprisingly, this increase in inspiratory driving pressure resulted in a significant increase in tidal volume and minute ventilation and as a result in a significant decrease in PaCO_2_.

**Figure 5:**
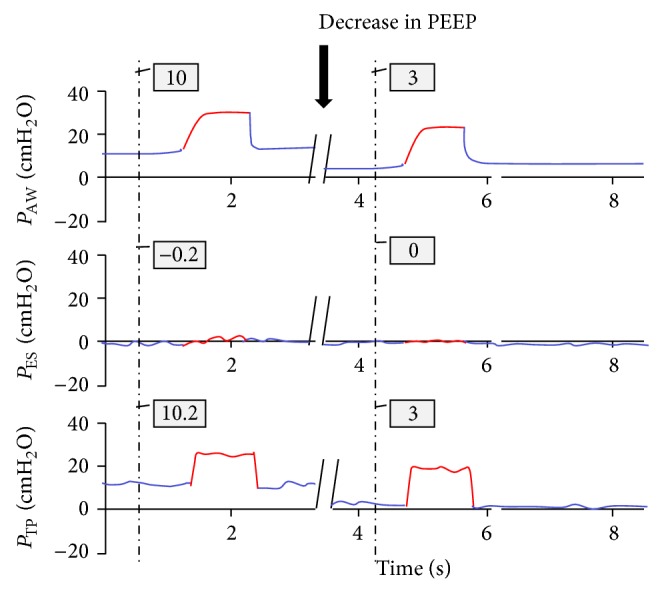
Representative pressure tracing of patient number 5. End expiratory transpulmonary (EETP) pressure is positive at 10.2 cm H_2_O. In this example, lowering PEEP closer towards zero decreased EETP pressure and resulted in a marked improvement in tidal volume and PaCO_2_. *P*_AW_: airway pressure, *P*_ES_: esophageal pressure, and *P*_TP_: transpulmonary pressure.

Interestingly, the last two patients (patients 5 and 6) whose PEEP was also determined by the ARDSnet guidelines were found to have a positive EETP pressure of 10.2 and 7.4 cm H_2_O, respectively. The positive EETP pressure in these two patients could be explained by inappropriately high PEEP values. Furthermore, once PEEP was lowered to a value that would result in EETP pressure that was close to zero, a significant improvement in gas exchange and tidal volume was noticed immediately (Figure 5). Tidal volumes per IBW increased from 3.21 and 3.8 mL/kg IBW to 6.25 and 6.76 mL/kg IBW, respectively (patients 5 and 6 in Table 2). Lowering inappropriately high PEEP to approximate pleural pressure resulted also in a considerable increase in cardiac output (patients 5 and 6 in Table 2).

Thus, in these patients with severe ARDS and poor lung compliance, exercising lung protective ventilation by following the ARDSnet guidelines with limitations on applied plateau pressure resulted in extremely low tidal volumes and consequently in severe respiratory acidosis. In these patients, in order not to exceed a plateau pressure of 30 cm H_2_O, setting up PEEP of 12 to 15 cm H_2_O left room for a limited inspiratory driving pressure of not more than 15 to 18 cm H_2_O.

Accordingly, while such inspiratory driving pressures would suffice most patients and would result in adequate alveolar ventilation with reasonable PaCO_2_ levels, following ARDSnet guidelines in these 6 patients with low respiratory system compliance has resulted in alveolar hypoventilation with extremely low tidal volumes (mean 3.96 ± 0.92 mL/kg IBW).

Severe hypercapnia with respiratory acidosis is associated and impaired right ventricular function and hemodynamics [18]. Thus, under normal circumstances, patients with such a severe hypercapnia would have been considered as candidates for extracorporeal removal of CO_2_ (such as pumpless extracorporeal lung assist, PECLA). These measures are invasive and necessitate the insertion of large bore indwelling intravascular catheters for vascular access. Such invasive measures for extracorporeal CO_2_ removal are associated with a significant rate of complications and include hemolysis, coagulation disorders, technical complications, and vascular complications such as compartment syndrome and leg ischemia [19–22].

Thus, the use of esophageal balloon with measurements of esophageal pressure as a surrogate for pleural pressure allowed us to better direct inspiratory driving pressure and PEEP and optimize them individually for each patient.

Furthermore, by assuming that a particular patient has high pleural pressure, one could argue that esophageal balloon use may be avoided simply by increasing inspiratory driving pressure in all patients with high PEEP and clinical suspicion of high pleural pressure. However such a generalized approach would theoretically overestimate actual pleural pressure in some patients, resulting in excessively high transpulmonary pressure. In the report of Talmor et al. [13], 3 out of 31 patients in the esophageal balloon group had high EETP pressure, and, in order to avoid high EETP pressure, PEEP had to be decreased in these 3 patients. Similarly, in our report in two out of six patients, pleural pressure was found to be unexpectedly low with positive EETP pressure. Without knowledge of the true EETP pressure, blindly increasing inspiratory driving pressure in these two patients would most likely have resulted in further increase in shunt fraction and decrease in cardiac output. Thus, the use of esophageal balloon in these two patients allowed us to correctly identify the existence of positive EETP pressure. The logical intervention of lowering PEEP to meet or approximate a zero EETP pressure resulted in a significant improvement in alveolar ventilation and cardiac output, without necessarily worsening oxygenation. In fact by lowering inappropriately high PEEP, P/F ratio has improved slightly from 130 to 135 mmHg and from 151 to 166 mmHg in patients 5 and 6, respectively.

There are a few limitations in this report. First is its size. However, it should be noticed that patients with severe ARDS and a concomitant severe respiratory acidosis to an extent reported in this small series are hard to come by. Secondly, this was not a comparative study. Ideally, two treatment modalities should have been compared, namely, extracorporeal removal of CO_2_ and esophageal balloon guided ventilation. However, such a comparative study would entail an enormous effort, possibly multicenter and international. Furthermore the feasibility of such a future study is questionable, since the availability of esophageal balloon and extracorporeal CO_2_ removal is still limited. Nevertheless, this report presents another treatment option that is less invasive, is easily accomplished where available, and, at least in the six patients in our report, averted the need for extracorporeal devices. There is no doubt that larger studies are needed to answer whether esophageal balloon guided mechanical ventilation is also associated with decreased mortality.

## 6. Conclusion

The use of esophageal balloon as a guide to mechanical ventilation may treat severe hypercapnia with severe respiratory acidosis in patients with ARDS and avert the need for extracorporeal removal of CO_2_.

## 7. Key Messages

- Esophageal balloon measurements may guide adjustments of mechanical ventilation in each patient, based on individual lung mechanics.
- Assessment of transpulmonary pressures may assist in averting severe hypercapnia.
- High plateau pressure is not necessarily associated with high transpulmonary pressure.

## Figures and Tables

**Table 1:** Characteristics of individual patients on ICU admission and prior to esophageal balloon insertion.

Patient and main diagnosis	Age/gender	APACHE II score/predicted mortality	Lung injury score^*^	PCO_2_	P/F ratio	PEEP	Number of failing organs during peak of disease	Total days on mechanical ventilation	28-day mortality
(1) Bilateral pneumonia + ARDS	49/F	33/60.1%	3.25	141	202	12	5	10	D
(2) Bilateral pneumonia + ARDS	67/M	40/91%	3.75	96	90	15	4	5	D
(3) Bilateral pneumonia + ARDS	84/F	29/67.2%	3.25	99	210	15	4	19	D
(4) Bilateral pneumonia + ARDS	76/M	29/68.7%	4	93	82.5	15	4	58	A
(5) Bilateral pneumonia + ARDS	40/F	27/63%	3.25	140	130	10	6	4	D
(6) Bilateral pneumonia + ARDS	81/M	29/67.2%	3.75	81	151	15	5	44	A

D: dead, A: alive.

^*^Lung injury severity score uses PaO_2_/FiO_2_ ratio, CXR, compliance of respiratory system, and level of PEEP. All are scored on a scale 0–4. Sum of scores is then divided by number of components. A total score greater than 2.5 defines ARDS.

**Table 2:** Respiratory parameters before and after intervention in individual patients.

Patient and main diagnosis	PEEP (cm H_2_O)		Inspiratory (driving) pressure		Plateau pressure		End inspiratory transpulmonary pressure		End expiratory transpulmonary pressure		Tidal volume/IBW		PaCO_2_ (mmHg)		Cardiac index (L/min/m^2^)	
	Before	After	Before	After	Before	After	Before	After	Before	After	Before	After	Before	After	Before	After
(1) Bilateral pneumonia + ARDS	12	12	15	25	27	36.5	16.6	19.5	2	0.1	2.61	5.23	141	58	3.27	3.05
(2) Bilateral pneumonia + ARDS	15	15	15	25	28.4	39.5	2.7	10	−1.7	−2	4.67	7.98	96	70	3.1	3.15
(3) Bilateral pneumonia + ARDS	15	15	15	25	28.7	38	9	18	−0.2	1	5	8.4	99	53	2.8	2.7
(4) Bilateral pneumonia + ARDS	15	15	15	25	28.5	35.3	7.6	12.4	−2.3	−1.7	4.5	7.85	93	60	3.76	3.86
(5) Bilateral pneumonia + ARDS	10	3	20	25	26	23.3	25.6	23	10.2	2.7	3.21	6.25	140	96	1.8	2.6
(6) Bilateral pneumonia + ARDS	15	5	20	20	34	24	20.6	17.7	7.4	1.1	3.8	6.76	81	50	3.2	3.95

**Table 3:** Respiratory and hemodynamic parameters on baseline and 1 hour after intervention guided by esophageal balloon measurements.

	Baseline before intervention	One hour after intervention	*P* value
PaCO_2_ (mmHg)	108.33 ± 25.65	64.5 ± 16.89	0.003
PH	7.01 ± 0.08	7.20 ± 0.08	<0.001
FiO_2_ (%)	66.66 ± 16.32	53.33 ± 5.16	0.08
PEEP	13.66 ± 2.16	10.83 ± 5.45	0.18
P/F ratio (mmHg)	144.25 ± 54.15	158.66 ± 30.11	0.45
Respiratory rate	23.7 ± 6.8	21.7 ± 4.3	0.3
Minute ventilation (L/min)	5.6 ± 1.8	9.1 ± 1.4	<0.001
Inspiratory pressure	16.66 ± 2.58	24.16 ± 2.04	0.007
EITP pressure	13.68 ± 8.69	16.76 ± 4.76	0.18
EETP pressure	1.5 ± 5.96	−0.25 ± 4.32	0.19
Plateau pressure	28.76 ± 2.77	32.76 ± 7.20	0.29
Tidal volume (in mL)	244.16 ± 69.88	435.0 ± 103.7	<0.001
Tidal volume (in mL/kg IBW)	3.96 ± 0.92	7.07 ± 1.21	<0.001
Total respiratory system compliance	16.56 ± 5.9	19.99 ± 5.73	0.11
Lung compliance^*^	23.09 ± 8.66	26.717 ± 9.67	0.29
Chest wall compliance^*^	40.5 ± 63.5	81.1 ± 124	0.168
Cardiac index (L/min/m^2^)	2.98 ± 0.66	3.21 ± 0.57	0.25

^*^Compliance in mL/cmH_2_O.

EITP: end inspiratory transpulmonary pressure, EETP: end expiratory transpulmonary pressure, and PEEP: positive end expiratory pressure.

## Abbreviations

ARDS: Acute respiratory distress syndrome
PEEP: Positive end expiratory pressure
EITP: End inspiratory transpulmonary pressure
EETP: End expiratory transpulmonary pressure
IBW: Ideal body weight.
